# Clinical Heterogeneity and Survival Disparities in Leiomyosarcoma: An Exploratory Single-Center Retrospective Observational Study

**DOI:** 10.3390/jcm15197693

**Published:** 2026-10-04

**Authors:** Rakesh Pinninti, Kiruthika Subramaniyan, Rajagopalan Iyer, Tasneem Rushdi, Harveen Kaur Gulati, Naga Prasanthi Akkineni, Srinath Gupta, Krishna Mohan Mallavarapu, Yashaswini Nallacheruvu, Sujit Chyau Patnaik, Santa Ayyagari, Suseela Kodandapani, Veeraiah Koppula, Deleep Gudipudi, Subramanyeshwar Rao Thammineedi, Senthil Rajappa

**Affiliations:** 1 Medical Oncology, Basavatarakam Indo American Cancer Hospital and Research Institute, Hyderabad 500082, India; 2 Surgical Oncology, Basavatarakam Indo American Cancer Hospital and Research Institute, Hyderabad 500082, India; 3 Radiation Oncology, Basavatarakam Indo American Cancer Hospital and Research Institute, Hyderabad 500082, India; 4 Pathology, Basavatarakam Indo American Cancer Hospital and Research Institute, Hyderabad 500082, India; 5 Radiodiagnosis, Basavatarakam Indo American Cancer Hospital and Research Institute, Hyderabad 500082, India

**Keywords:** leiomyosarcoma, soft tissue sarcoma, anatomical site, overall survival, metastatic disease

## Abstract

**Background:** Leiomyosarcoma (LMS) is a rare aggressive sarcoma with substantial heterogeneity in anatomical presentation, stage, treatment, and survival. We evaluated clinical characteristics, treatment patterns, and survival, with particular emphasis on whether anatomical site independently predicted overall survival (OS). **Methods:** We retrospectively reviewed 240 adults with histologically confirmed LMS diagnosed between January 2015 and May 2026. OS was calculated from diagnosis to death or last contact, with administrative censoring on 1 July 2026. Kaplan–Meier and Cox proportional-hazards methods were used to evaluate age, sex, anatomical site, histologic grade, and disease stage. Multivariable models adjusted for age, sex, grade, and stage assessed the independent association of anatomical site with OS. **Results:** Median age was 56 years, and 152 patients (63.3%) were female. Disease was localized in 120 (50.0%), de novo metastatic in 53 (22.1%), recurrent metastatic in 35 (14.6%), and recurrent localized in 12 (5.0%). Median OS was 37.0 months. Localized disease was associated with substantially longer OS than metastatic disease (61.0 vs. 13.0 months; *p* < 0.001). Grade 3 disease was associated with shorter OS than grade 1/2 disease (25.6 vs. 61.1 months; *p* < 0.001). In multivariable analyses, metastatic disease remained strongly associated with inferior OS (HR, 5.59–5.94; *p* < 0.001), as did age ≥ 50 years (HR, 1.86–1.92; *p* = 0.006–0.004) and grade 3 histology (HR, 1.74–1.92; *p* = 0.018–0.007). Anatomical site was not independently associated with OS (extremity/trunk vs. non-extremity: HR, 1.60; *p* = 0.063; non-uterine vs. uterine: HR, 1.23; *p* = 0.41). **Conclusions:** Disease stage was the strongest prognostic factor, while older age and grade 3 histology were independently associated with poorer OS. Although extremity/trunk LMS showed better univariable survival, anatomical site was not independently associated with OS after adjustment.

## 1. Introduction

Leiomyosarcoma (LMS) is a rare, aggressive malignant neoplasm of smooth-muscle origin [1,2]. It can arise in multiple anatomical locations, including the uterus, retroperitoneum, intra-abdominal soft tissues, extremities, trunk, and other visceral or vascular sites [3,4,5]. This anatomical diversity is accompanied by marked variation in clinical presentation, resectability, metastatic pattern, treatment strategy, and survival [6,7,8].

The heterogeneity of LMS complicates the interpretation of outcome differences across anatomical sites [6,9]. Published evidence frequently consists of site-specific series, such as uterine or retroperitoneal cohorts, in which differences in disease stage, tumor burden, resectability, and treatment selection may coexist with differences in tumor biology [5,6,7,10,11,12]. Consequently, an apparent survival difference by anatomical origin does not necessarily reflect an independent effect of site.

Management of localized LMS is centered on complete surgical resection, while the use of neoadjuvant or adjuvant chemotherapy and radiotherapy is individualized according to anatomical site, tumor characteristics, margin status, recurrence risk, and multidisciplinary assessment [13]. In advanced disease, systemic therapy is the principal treatment modality, with local treatments such as radiotherapy or metastasectomy used selectively [13]. Real-world treatment patterns therefore vary substantially across patients.

Indian real-world datasets describing LMS remain limited, with available evidence largely derived from single-institution LMS-specific cohorts or broader soft-tissue sarcoma series [14,15,16]. Much of the published literature on LMS has focused on individual anatomical sites, limiting direct assessment of whether anatomical site is independently associated with survival after accounting for disease stage. A single-institution mixed-site cohort that simultaneously examines stage, site, metastatic pattern, treatment variability, and survival can provide a more integrated view of the clinical factors associated with observed outcome disparities. We therefore evaluated the clinical characteristics and survival outcomes of patients with LMS, the associations of disease stage and anatomical site with overall survival, and whether site-associated differences persisted after adjustment for disease stage and other available clinical factors.

## 2. Methods

### 2.1. Study Design and Setting

This was a retrospective, single-centre observational cohort study conducted at a tertiary cancer centre, Hyderabad, India. Adult patients with histologically confirmed LMS diagnosed at the institution between January 2015 and May 2026 were identified from electronic health records and an institutional structured registry. The study was approved by the Institutional Ethics Committee, with a waiver of informed consent granted for the retrospective review of medical records.

### 2.2. Patient Population and Disease Classification

Patients aged 18 years or older with histologically confirmed LMS were eligible. Disease status at initial institutional assessment was classified as localized, de novo metastatic, recurrent localized, or recurrent metastatic disease. For the principal stage comparison, early/localized disease comprised localized and recurrent localized disease, whereas metastatic disease comprised de novo and recurrent metastatic disease. Primary site was classified as uterus, non-uterine abdomen, extremity/trunk, retroperitoneum, or head and neck. Anatomical site was evaluated using two comparisons: extremity/trunk versus non-extremity and uterine versus non-uterine. These analyses were considered part of the overall site-based survival assessment.

### 2.3. Data Collection

Clinical data were abstracted into a structured registry. Variables included age, sex, ECOG performance status, tumor size, histologic grade, nodal status, molecular findings where available, primary site, disease stage, surgery, surgical intent and margin status, neoadjuvant chemotherapy and radiotherapy, adjuvant chemotherapy and radiotherapy, recurrence, metastatic sites, oligometastatic disease, systemic therapy, number of systemic treatment lines, first-line regimen and response, metastasectomy, subsequent systemic therapy, maintenance treatment, and follow-up. Treatment exposures were recorded as delivered in routine clinical practice. Therapy comparisons were considered descriptive or exploratory. For patients in whom histologic grade was unavailable or incomplete, archived histopathology blocks were re-reviewed by pathologists who did not have access to patients’ clinical outcomes or survival data.

### 2.4. Outcome

The primary outcome was overall survival (OS). The study evaluated the clinical characteristics and survival outcomes of patients with LMS, with particular emphasis on the associations of disease stage and anatomical site with overall survival, including comparisons of extremity/trunk versus non-extremity LMS and uterine versus non-uterine LMS, and on whether these associations persisted after adjustment for other available clinical factors. OS was defined as time from date of diagnosis to death from any cause. Patients alive at last contact were censored at the earlier of their last documented follow-up date or the administrative cutoff of 1 July 2026.

Secondary outcomes included disease-stage distribution, primary-site distribution, recurrence, metastatic patterns, treatment utilization, and survival by age group and by the two anatomical-site comparisons.

### 2.5. Statistical Analysis

Continuous variables were summarized using medians and ranges, and categorical variables using frequencies and percentages. Age was categorized as <50 versus ≥50 years for clinical interpretability. OS was estimated using the Kaplan–Meier method and compared between groups using the log-rank test. Median potential follow-up was estimated using reverse Kaplan–Meier analysis.

Univariable survival comparisons were performed for age group (≥50 vs. <50 years), sex, the two anatomical-site comparisons (extremity/trunk vs. non-extremity; uterine vs. non-uterine), histologic grade, and disease stage (early/localized vs. metastatic). Because the number of grade 1 tumors was modest (*n* = 16), histologic grade was dichotomized as grade 3 versus grade 1/2 combined for the primary comparison. Tumor size was evaluated univariably both as a binary variable (≥5 cm vs. <5 cm) and as a continuous covariate (per cm), using the log-rank test and Cox proportional-hazards regression, respectively, restricted to the subset of patients with a recorded tumor size.

Multivariable Cox proportional-hazards regression was used to determine whether the observed associations persisted after adjustment. The extremity-site model included age group, sex, histologic grade (grade 3 vs. grade 1/2), metastatic versus early stage, and non-extremity versus extremity site. The uterine-site model included the same covariates, substituting non-uterine versus uterine site for the site. The extremity and uterine comparisons were modelled separately because uterine primaries are a subset of non-extremity primaries. Hazard ratios (HRs) with 95% confidence intervals (CIs) were reported. As a sensitivity analysis, age was additionally modelled as a continuous covariate in place of the age ≥ 50/<50 categories, using the same Cox proportional-hazards framework. Two-sided values of *p* < 0.05 were considered statistically significant.

Margin status and nodal status were structurally unavailable for unresected patients and could not be incorporated into an all-cohort model without restricting the analysis to surgically resected patients; these variables were therefore excluded from the multivariable models. Accordingly, multivariable models included age, sex, grade, stage, and anatomical site, with complete-case analysis for each model. Statistical analysis was performed using IBM SPSS version 22.0 software (New York, NY, USA) and Python (lifelines package, v0.30.3).

## 3. Results

### 3.1. Patient Characteristics

A total of 240 patients were evaluable for the current analysis. Median age was 56 years (range, 27–86), and 152 patients (63.3%) were female. Disease at presentation was localized in 120 patients (50.0%), de novo metastatic in 53 (22.1%), recurrent metastatic in 35 (14.6%), and recurrent localized in 12 (5.0%); 20 patients (8.3%) were not classifiable by stage. The most frequent primary sites were extremity/trunk in 67 patients (27.9%), uterus in 55 (22.9%), retroperitoneum in 54 (22.5%), non-uterine abdomen in 50 (20.8%), and head and neck in 13 (5.4%). Baseline demographic and disease characteristics of the entire cohort are listed in Table 1.

### 3.2. Treatment Patterns and Clinical Course

Among 132 patients classified as early/localized disease, surgery was performed in 119 of 127 evaluable patients (93.7%). Neoadjuvant chemotherapy was administered to 6 of 118 evaluable patients (5.1%), adjuvant chemotherapy to 32 of 112 (28.6%), and adjuvant radiotherapy to 26 of 107 (24.3%). Recurrence was recorded in 40 of 93 evaluable patients (43.0%). Supplementary Table S1 enumerates treatment patterns as per baseline disease stage.

ECOG performance status at diagnosis was evaluable in 235 of 240 patients (97.9%); the great majority (231/235, 98.3%) were coded as performance status 0–1 (fit for standard treatment), with only 4 patients (1.7%) coded as performance status 3–4. Among the 164 patients who underwent surgery, margin status was recorded in 136 (82.9%): resection was R0 in 133 (97.8%), R1 in 1 (0.7%), and R2 in 2 (1.5%). Among 88 patients with metastatic disease, systemic therapy was administered to 67 of 79 evaluable patients (84.8%), while metastasectomy was performed in 9 of 68 evaluable patients (13.2%). Among patients with metastatic disease, the most frequent metastatic sites were lung (62.2%), lymph node (37.8%), liver (34.1%), peritoneum (32.9%), and bone (24.4%) (Supplementary Figure S1).

### 3.3. Overall Survival

There were 131 deaths among 240 evaluable patients (54.6%). Median OS for the whole cohort was 37.0 months (95% CI, 26.0–48.0). Median potential follow-up, estimated using the reverse Kaplan–Meier method, was 43.0 months. Among the 109 patients censored at last follow-up, the median observed follow-up was 32.0 months (IQR, 16.0–50.0; range, 0–120 months). Table 2 summarizes OS according to disease stage, primary site, age group, and histologic grade.

### 3.4. Survival According to Disease Status

Early/localized disease had a median OS of 61.0 months (95% CI, 43.9–78.1), whereas metastatic disease had a median OS of 13.0 months (95% CI, 10.2–15.8; log-rank *p* < 0.001) (Figure 1). To assess whether combining localized and recurrent localized disease influenced the primary stage comparison, we performed a sensitivity analysis in which strictly localized, recurrent localized, and metastatic disease were modeled separately. In univariable analysis, recurrent localized disease was not significantly associated with OS compared with strictly localized disease (HR, 1.32; 95% CI, 0.56–3.13; *p* = 0.522), whereas metastatic disease was strongly associated with inferior OS (HR, 5.15; 95% CI, 3.42–7.76; *p* < 0.001). This pattern was preserved after multivariable adjustment: recurrent localized disease was not significantly associated with OS compared with strictly localized disease in either the extremity/trunk model (HR, 1.54; 95% CI, 0.63–3.76; *p* = 0.344) or the uterine model (HR, 1.34; 95% CI, 0.55–3.24; *p* = 0.521), while metastatic disease remained strongly associated with inferior OS (HR, 5.75; 95% CI, 3.60–9.19; *p* < 0.001, and HR, 6.12; 95% CI, 3.83–9.78; *p* < 0.001, respectively).

### 3.5. Survival According to Anatomical Site

Patients with extremity/trunk primaries had longer OS than those with non-extremity primaries, with a median OS of 53.0 months (95% CI, 22.4–83.6) versus 28.0 months (95% CI, 17.0–39.0), respectively (log-rank *p* = 0.040) (Figure 2). In the uterine versus non-uterine comparison, median OS was 28.0 months (95% CI, 12.3–43.7) among patients with uterine LMS and 39.0 months (95% CI, 22.4–55.6) among those with non-uterine LMS; this difference was not statistically significant (log-rank *p* = 0.401) (Supplementary Figure S2).

Metastatic disease was more frequent among uterine than non-uterine LMS (28/52 [54%] vs. 60/168 [36%]). This stage imbalance was statistically significant (χ^2^ = 4.71, *p* = 0.030), whereas the corresponding imbalance between extremity/trunk and non-extremity primaries (32.3% vs. 43.0% metastatic) did not reach significance (*p* = 0.19) (Supplementary Table S2). Supplementary Figure S3 shows the distribution of disease stage across primary anatomical sites.

### 3.6. Survival According to Histologic Grade

Histologic grade was available for 216 of 240 patients (90.0%), 16 grade 1 (7.4%), 65 grade 2 (30.1%), and 135 grade 3 (62.5%) (Table 1). Given the modest number of grade 1 tumors, grade was analyzed as grade 3 versus grade 1/2 combined for the primary comparison.

Across the full grade-evaluable cohort, OS differed significantly by grade: patients with grade 3 tumors had a median OS of 25.6 months (95% CI, 18.1–37.4) and a 5-year OS of 28.0% (95% CI, 18.8–37.9%), compared with 61.1 months (95% CI, 52.3–not reached) and 54.5% (95% CI, 38.2–68.1%) for patients with grade 1/2 tumors (log-rank *p* < 0.001; Figure 3).

### 3.7. Survival According to Tumor Size

Tumor size was evaluable in 204 of 240 patients (85.0%). Tumor size was not associated with OS, whether evaluated as a binary variable using a 5 cm threshold (≥5 cm, *n* = 163, 106 events, median OS 37.6 months [95% CI, 27.6–50.8] vs. <5 cm, *n* = 41, 20 events, median OS 58.1 months [95% CI, 12.8–not reached]; log-rank *p* = 0.94) or as a continuous covariate in a univariable Cox model (HR, 1.02 per cm; 95% CI, 0.99–1.06; *p* = 0.15). Given the absence of a univariable association, tumor size was not carried into the multivariable models. Among the 88 patients with metastatic disease (Table 2), the number and distribution of metastatic sites were evaluable in 79 (89.8%); 6 of these (7.6%) had oligometastatic disease (disease confined to one organ with fewer than four lesions), and 73 (92.4%) had polymetastatic disease. Patients with oligometastatic disease had substantially longer OS than those with polymetastatic disease (median OS not reached [95% CI, 4.4 months–not reached] vs. 12.5 months [95% CI, 9.6–19.7]; log-rank *p* = 0.023; Cox HR, 0.22; 95% CI, 0.05–0.92; *p* = 0.037).

Comparing patients registered in 2015–2020 (*n* = 89) with those in 2021–2026 (*n* = 151), unadjusted median OS was 44.9 months versus 25.5 months, respectively (log-rank *p* = 0.13), with no statistically significant difference between eras. The observed difference may partly reflect shorter follow-up in the more recent era. A sensitivity analysis excluding the most recent, follow-up-limited patients (2024–2026) substantially attenuated this difference (HR, 1.46 vs. 1.69, no longer significant). Overall, these data do not provide evidence of a statistically significant era effect on survival in this cohort.

### 3.8. Survival According to Treatment Variables

Among 164 patients who underwent surgery, margin status was available for 136 (82.9%); 133 (97.8%) had R0 resection, 1 (0.7%) had R1 resection, and 2 (1.5%) had R2 resection. Because only three patients had non-R0 resection, an R0 versus R1/R2 survival comparison was considered insufficiently informative for reliable inferential analysis.

Among patients with early/localized disease and known adjuvant treatment status, exploratory survival analyses were performed according to receipt of adjuvant chemotherapy and radiotherapy. Patients who received adjuvant chemotherapy had a median OS of 52.8 months (95% CI, 28.0–not reached), compared with 66.0 months (95% CI, 52.3–not reached) among those who did not receive chemotherapy (HR, 1.35; 95% CI, 0.68–2.66; log-rank *p* = 0.39). For adjuvant radiotherapy, median OS was not reached among patients who received radiotherapy compared with 61.1 months (95% CI, 43.9–97.4) among those who did not (HR, 0.71; 95% CI, 0.31–1.65; log-rank *p* = 0.43).

In stage-stratified treatment-inclusive sensitivity analyses, surgery was independently associated with longer OS in early/localized disease (HR, 0.11–0.13; *p* = 0.016–0.022), whereas adjuvant chemotherapy and radiotherapy were not independently associated with OS. In metastatic disease, metastasectomy was independently associated with longer OS (HR, 0.21–0.25; *p* = 0.039–0.043), while surgery and systemic therapy were not independently associated with OS; anatomical site was not independently associated with OS in either disease stratum (see Supplementary Analysis S1, Supplementary Tables S5 and S6). In an early/localized disease sensitivity model excluding surgery, grade 3 histology was independently associated with inferior OS (HR, 2.80; *p* = 0.039 in the extremity-site model; HR, 2.83; *p* = 0.024 in the uterine-site model), whereas this association was attenuated when surgery was included (see Supplementary Analysis S1, Supplementary Tables S5 and S6).

### 3.9. Multivariable Analysis of Anatomical Site

The extremity and uterine comparisons were modelled separately because uterine primaries are a subset of non-extremity primaries; combining them in a single model would compare overlapping groups rather than independent ones. Histologic grade (grade 3 vs. grade 1/2) was included as a covariate in both models alongside age group, sex, and stage.

In the extremity-site model, non-extremity versus extremity/trunk primary site was not independently associated with OS (HR, 1.60; 95% CI, 0.97–2.63; *p* = 0.063). Similarly, in the uterine-site model, non-uterine versus uterine primary site was not independently associated with OS (HR, 1.23; 95% CI, 0.75–2.03; *p* = 0.41). Metastatic disease remained strongly associated with inferior OS in both models (HR, 5.59; 95% CI, 3.55–8.80; *p* < 0.001 in the extremity-site model and HR, 5.94; 95% CI, 3.79–9.33; *p* < 0.001 in the uterine-site model). Age ≥ 50 years was independently associated with worse OS in both models (HR, 1.86; 95% CI, 1.19–2.91; *p* = 0.006 and HR, 1.92; 95% CI, 1.23–3.01; *p* = 0.004, respectively), as was grade 3 histology (HR, 1.92; 95% CI, 1.20–3.09; *p* = 0.007 and HR, 1.74; 95% CI, 1.10–2.76; *p* = 0.018, respectively). Gender was not independently associated with OS in either model (male vs. female HR, 1.09; 95% CI, 0.70–1.71; *p* = 0.70 and HR, 0.93; 95% CI, 0.58–1.49; *p* = 0.75, respectively). In a sensitivity analysis modeling age as a continuous variable, age remained independently associated with OS (HR, 1.035 per year; 95% CI, 1.016–1.054; *p* < 0.001 in the extremity-site model and HR, 1.034 per year; 95% CI, 1.016–1.053; *p* < 0.001 in the uterine-site model). Metastatic disease (HR, 6.35; 95% CI, 3.96–10.18; *p* < 0.001 and HR, 6.66; 95% CI, 4.18–10.63; *p* < 0.001, respectively) and grade 3 histology (HR, 1.93; 95% CI, 1.21–3.08; *p* = 0.006 and HR, 1.79; 95% CI, 1.13–2.82; *p* = 0.013, respectively) remained independently associated with inferior OS when age was modeled continuously (Supplementary Table S3). Figure 4 and Table 3 present the multivariable Cox models evaluating the association between anatomical site and overall survival.

## 4. Discussion

This real-world cohort demonstrates substantial variability in the clinical profile and treatment of LMS, with patients presenting across multiple anatomical sites and a broad spectrum of disease extent. The central finding is that disease stage was the strongest independent predictor of OS. Although recurrent localized disease may represent a clinically distinct disease state, sensitivity analysis separating recurrent localized from strictly localized disease did not materially alter the association between metastatic disease and OS, supporting the use of the combined early/localized category for the primary analysis.

Anatomical site showed different patterns in the univariable analyses. Extremity/trunk LMS had longer OS than non-extremity LMS, whereas OS did not differ significantly between uterine and non-uterine LMS. Metastatic disease was more frequent among uterine than non-uterine LMS, whereas the difference in metastatic disease rates between extremity/trunk and non-extremity LMS was not statistically significant. Although extremity/trunk primaries had longer OS on univariable analysis, this association was no longer statistically significant after adjustment for stage, age, histologic grade, and sex. Neither anatomical-site comparison was independently associated with OS in the multivariable models.

Age also merits consideration. Using a cutoff of 50 years, age ≥ 50 years was significantly associated with shorter OS on univariable analysis and remained independently associated with worse OS in both multivariable models. The continuous-age sensitivity analysis further supported the observed age effect. When age was modeled continuously, each additional year of age was associated with a 3.5% increase in the hazard of death per additional year in the extremity-site model (HR, 1.035; 95% CI, 1.016–1.054; *p* < 0.001) and a 3.4% increase in the uterine-site model (HR, 1.034; 95% CI, 1.016–1.053; *p* < 0.001).

The treatment data demonstrate substantial real-world variability. Surgery was performed in most evaluable patients with early/localized disease, whereas adjuvant chemotherapy and radiotherapy were used selectively. Most evaluable patients with metastatic disease received systemic therapy, while metastasectomy was performed in a selected minority. In a retrospective cohort, adjuvant treatment is not randomly assigned and may preferentially be administered to patients perceived to have higher-risk disease. Such confounding by treatment selection may therefore contribute to the observed survival difference. In particular, the numerically shorter OS observed in our cohort among patients receiving adjuvant chemotherapy should not be interpreted as evidence of treatment-associated harm, as treatment allocation was non-random and may have been influenced by underlying recurrence risk and other clinicopathological factors (confounding by indication). For this reason, treatment exposures are reported descriptively (Results; Supplementary Table S1) rather than as covariates in the adjusted survival models, consistent with the study’s aim of isolating the independent associations of stage, site, age, sex, and grade with OS. Treatment variables were not incorporated as covariates into the primary multivariable Cox models; treatment-associated survival analyses were performed separately as exploratory analyses (see Supplementary Analysis S1, Supplementary Tables S5 and S6). The primary Cox model evaluated baseline prognostic factors and should therefore be interpreted as a baseline prognostic model rather than a treatment-adjusted model. Differences in study design complicate direct comparison with published series. Much of the available LMS literature consists of site-specific cohorts, large registry analyses focused on individual anatomical sites, or selective resection series that may underrepresent unresectable and metastatic disease [4,5,6,7,8,10,11,12,17,18]. For extremity LMS, a 1528-patient SEER series reported a five-year OS of 61.7%, and the authors state that extremity LMS tends to have a better prognosis than uterine, retroperitoneal, or major-vessel LMS [5]. Our extremity/trunk subgroup had a five-year OS of 47.0% (95% CI, 30.9–61.5%), which was longer than that observed among patients with non-extremity disease (five-year OS, 29.0% [95% CI, 20.2–38.4%]), and the univariate log-rank comparison (*p* = 0.040) would, taken alone, appear to support the same conclusion. However, the apparent survival advantage was no longer statistically significant after adjustment for age, sex, histologic grade, and stage (HR, 1.60; 95% CI, 0.97–2.63; *p* = 0.063). The longer survival observed for extremity LMS in registry-based comparisons may, at least in part, reflect differences in disease stage rather than an independent association with anatomical site. This interpretation remains hypothesis-generating, particularly given the observational design and potential for residual confounding.

For retroperitoneal LMS, an MD Anderson series of 85 completely resected patients reported a median OS of 8.3 years (95% CI, 5.7–12.3), while a separate SEER analysis reported a five-year OS of 40% [8,18]. In this analysis, we pooled retroperitoneal patients within the broader non-extremity/non-uterine group rather than reporting them separately.

Uterine LMS remains associated with highly stage-dependent outcomes, with reported 5-year survival ranging from approximately 25% to 76% and falling to 10–15% among patients presenting with metastatic disease [19,20,21]. In our uterine LMS subgroup, the overall 5-year OS was 30.8% (95% CI, 16.8–46.0%), compared with 54.7% among patients with early/localized disease (median OS, 71 months) and 12.1% among those with metastatic disease (median OS, 20 months), broadly consistent with published experience.

As noted in our cohort, metastatic disease was more frequent among uterine than non-uterine LMS (28/52 [54%] vs. 60/168 [36%], χ^2^ = 4.71, *p* = 0.030). This observation is broadly consistent with prior mixed-site LMS data showing differences in baseline tumor characteristics across anatomical sites, including more advanced and higher-grade disease in uterine and retroperitoneal LMS compared with extremity or trunk tumors, without an independent difference in disease-specific survival by site [6].

A large SEER analysis of 1396 patients with uterine LMS reported five-year disease-specific survival of 91.4%, 74.2%, and 57.4% for grade 1, 2, and 3 tumors, respectively; however, grade was available for only 804 patients (57.6%), and the uterine-only cohort could not establish whether the prognostic effect of grade was independent of anatomical site in a multi-site LMS population [11]. In the present cohort, grade is evaluable in 90.0% of patients across the full spectrum of stage and treatment status. In multivariable models adjusted for age, sex, site, and stage, grade 3 histology was independently associated with an approximately two-fold higher hazard of death relative to grade 1/2 (HR, 1.92 and 1.74 in the extremity- and uterine-site models, respectively), providing a multi-site, stage-adjusted evaluation that a uterine-only cohort could not offer.

A recent, large-scale analysis of 11,751 patients with LMS from the US SEER registry (2005–2022; Oswald et al., J Natl Cancer Inst, 2026) provides the most rigorous multi-site characterization of LMS survival to date [22]. In that cohort, a multivariable model stratified by stage and age identified anatomical site, histologic grade, race, marital status, and household income as independently associated with disease-specific survival: Black patients, widowed patients, and those with the lowest household income all had significantly shorter survival, alongside site itself. Notably, in that dataset race and site are themselves inter-connected: uterine tumors accounted for a significantly greater proportion of cases in Black patients, while the skin and limb were significantly more common sites in the white population; and even within the same anatomical site, Black patients had consistently poorer survival than white patients (for example, 5-year survival in uterine LMS was 34.1% in Black patients versus 43.6% in white patients) [22].

Two observations from this SEER analysis are directly relevant to interpreting the present cohort. First, because site, race, marital status, and income are all independently prognostic in a single US model, anatomical-site risk stratification derived from US registry data is not free of the specific social and healthcare-access scenario; for example, insurance-linked access to specialist sarcoma care, documented under-representation of Black patients in sarcoma clinical trials, and race-correlated differences in stage at presentation are all plausible contributors to the observed site-associated survival differences. The sensitivity analysis in Oswald et al., in which exclusion of grade altered the sites reaching independent significance, further illustrates that site estimates may be sensitive to model specification. Our cohort is drawn from a single tertiary hospital in a low- and middle-income country (LMIC) setting with a comparatively racially homogeneous population and a single, non-insurance-stratified referral pathway. Our single-centre cohort differs substantially from the population-based SEER dataset in patient composition, healthcare setting, and availability of clinical variables. Differences in case mix, referral patterns, and unmeasured socioeconomic factors may therefore contribute to the differing site-associated estimates. This discrepancy may partly reflect differences between population-based registries, which offer greater power but limited clinical granularity, and hospital-based cohorts, which provide richer clinical characterization but are smaller and more susceptible to selection bias.

### Limitations

This study should be interpreted in the context of several limitations inherent to its design, cohort composition, and available data. First, the retrospective single-centre design introduces potential selection and information bias. Second, the cohort spans January 2015 to May 2026, during which staging practices, imaging availability, treatment options, referral pathways, and multidisciplinary decision-making may have changed, although no statistically significant era effect on survival was observed. Third, event numbers in some anatomical subgroups were small (e.g., extremity/trunk, *n* = 67), resulting in wide confidence intervals for the site comparisons. Fourth, missing data affected several key variables to varying degrees, summarized in Table 4. Stage was unclassifiable in 20 of 240 patients (8.3%); tumor size was missing in 36 of 240 (15.0%); and histologic grade was missing in 24 of 240 (10.0%). Among treatment variables, missingness ranged from 3.8% (surgery status among early/localized patients) to 29.5% (recurrence status among early/localized patients) and 22.7% (metastasectomy status among metastatic patients). Grade and tumor size missingness were addressed by complete-case analysis in the multivariable models (*N* = 197). Fifth, margin status and nodal status remain structurally unavailable for unresected patients and could not be incorporated into an all-cohort model without restricting the analysis to surgically resected patients; tumor size was not associated with OS in univariable analysis and was excluded from the multivariable models. Finally, as detailed above, treatment variables were not included in the baseline prognostic survival models because treatment assignment is itself downstream of stage and clinician judgement in this retrospective setting. Despite these limitations, the study provides an integrated real-world assessment of clinical heterogeneity and survival disparity in LMS.

## 5. Conclusions

LMS demonstrates marked variability in anatomical presentation, disease stage, metastatic pattern, and treatment exposure. In this cohort, disease stage was the strongest independent predictor of OS, with metastatic disease associated with approximately fivefold to sixfold higher hazard of death than early/localized disease. Older age was also independently associated with inferior OS. Although extremity/trunk primaries were associated with longer OS on univariable analysis, neither extremity/trunk nor uterine primary site was independently associated with OS after adjustment for age, sex, histologic grade, and disease stage.

## Figures and Tables

**Figure 1:**
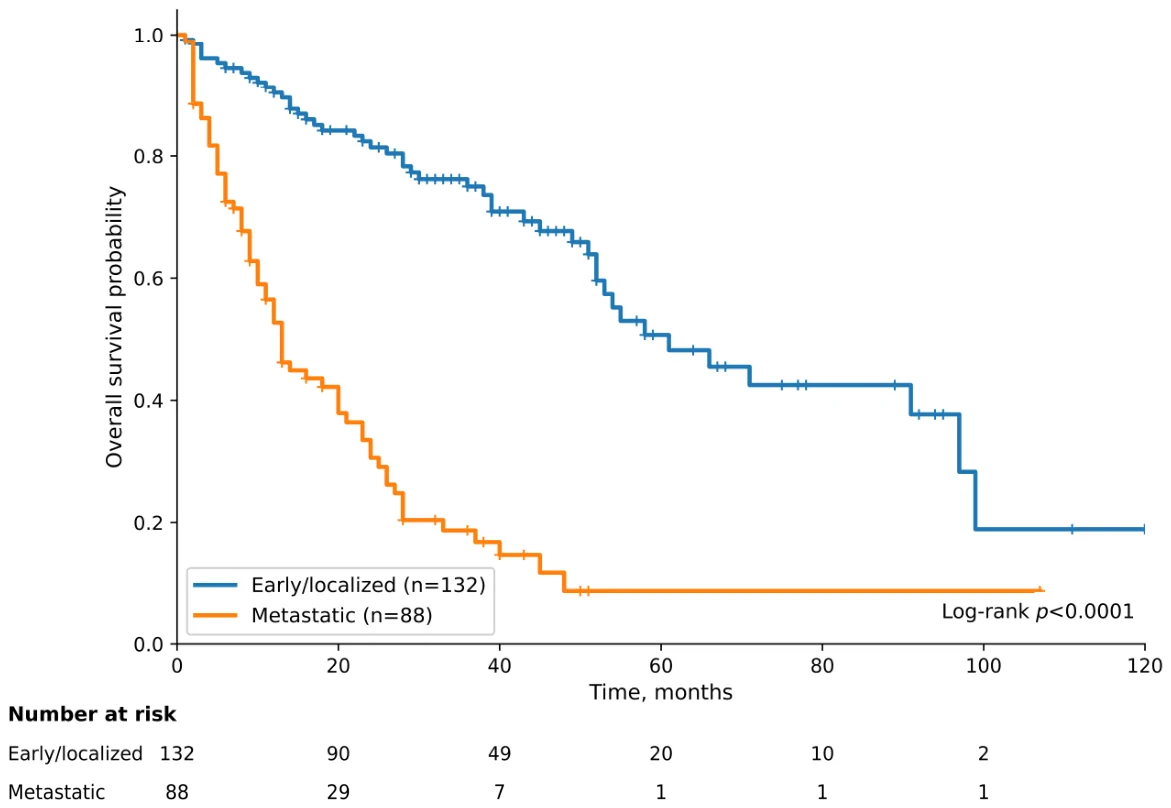
Kaplan–Meier overall survival according to early/localized versus metastatic disease.

**Figure 2:**
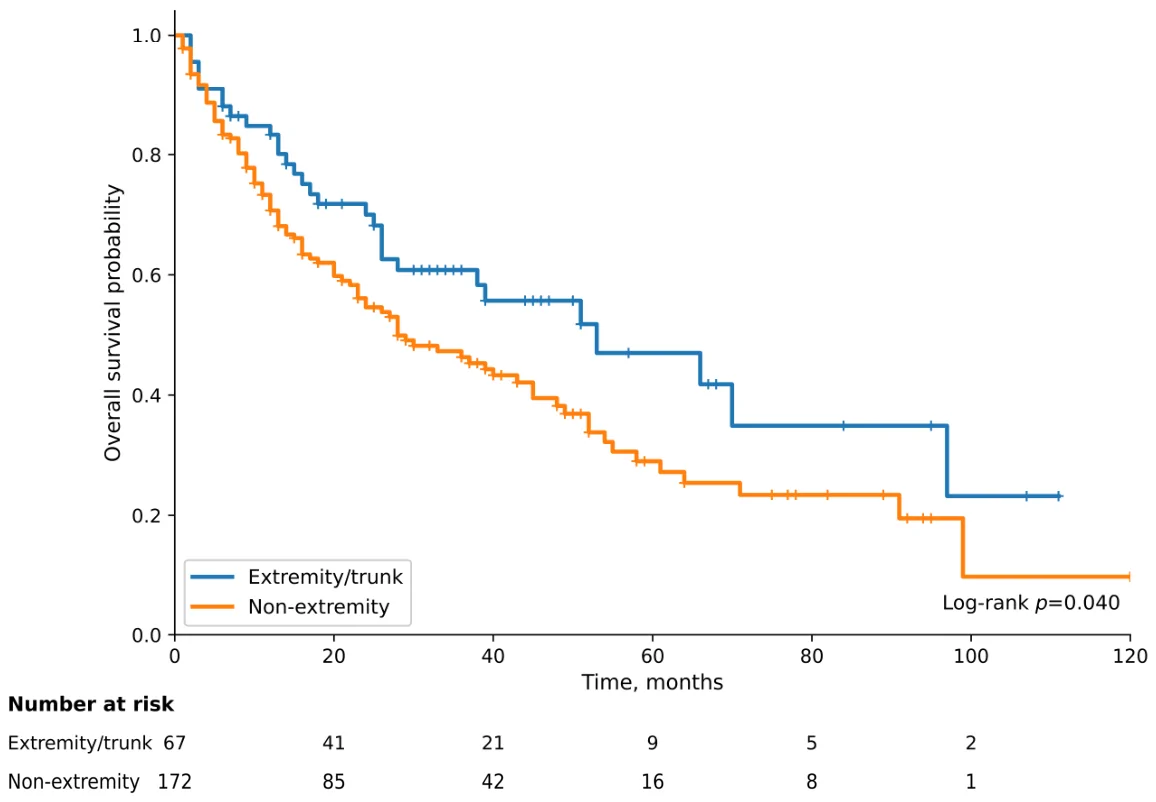
Kaplan–Meier overall survival according to extremity/trunk versus non-extremity primary site.

**Figure 3:**
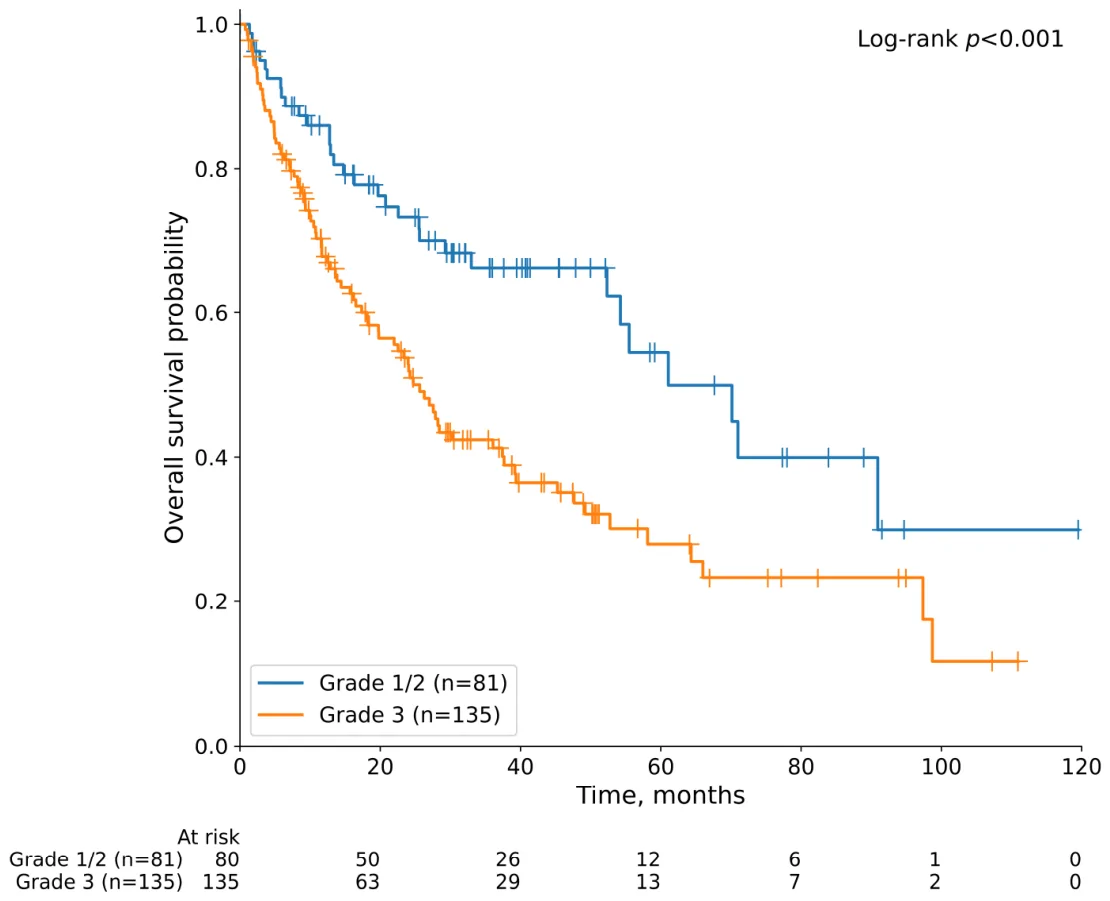
Kaplan–Meier overall survival according to histologic grade.

**Figure 4:**
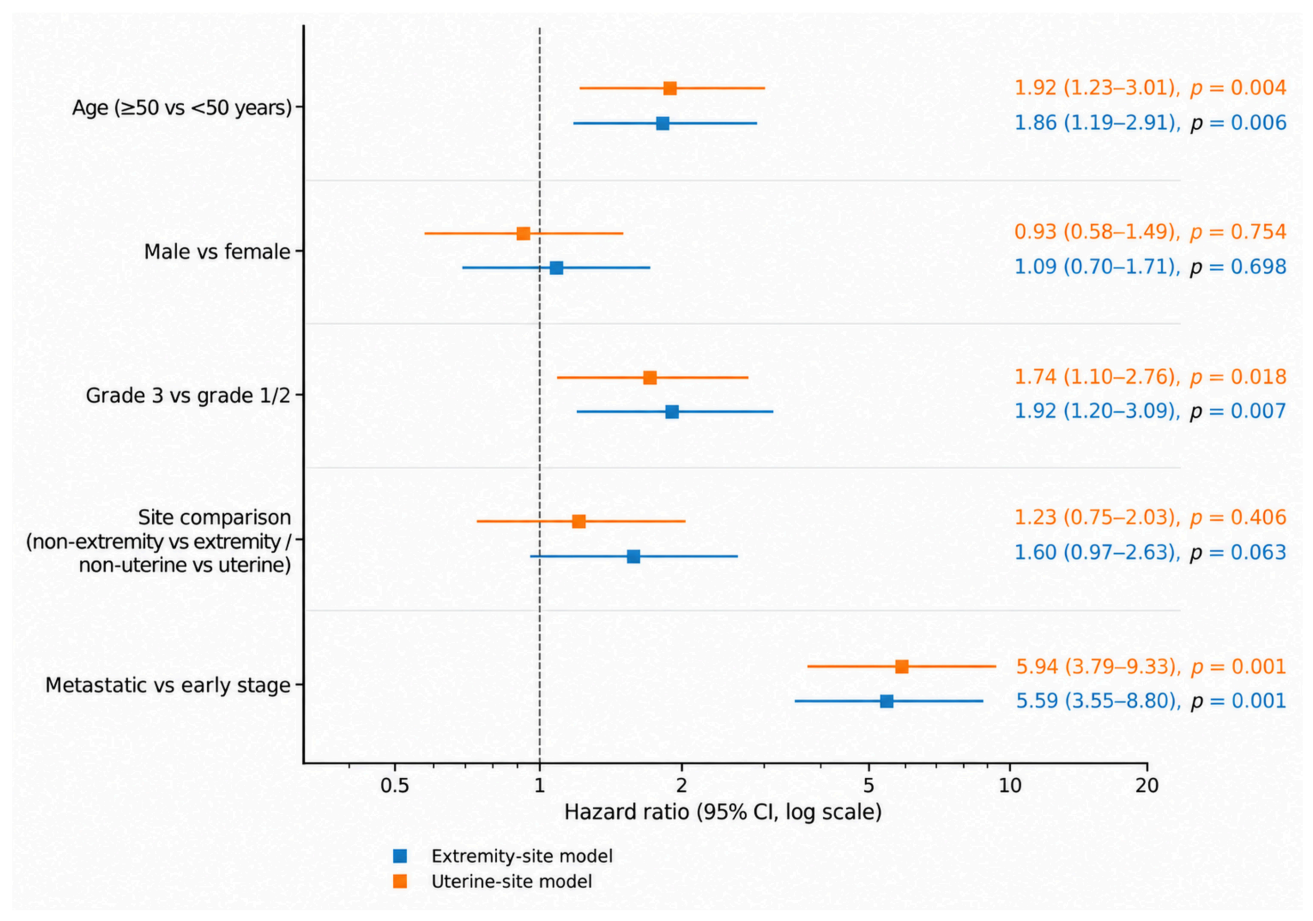
Forest plot of two parallel multivariable Cox proportional hazards models evaluating the association of anatomical site with overall survival. Both models were adjusted for age group, sex, histologic grade, and disease stage.

**Table 1:** Baseline demographic and disease characteristics of the entire cohort (*n* = 240).

Characteristic	Category	*n* (%)
Age, years	Median (range)	56 (27–86)
Age group	<50 years	78 (32.5%)
	≥50 years	162 (67.5%)
Sex	Female	152 (63.3%)
	Male	88 (36.7%)
Disease status	Localized	120 (50.0%)
	Recurrent localized	12 (5.0%)
	Recurrent metastatic	35 (14.6%)
	De novo metastatic	53 (22.1%)
	Unknown/not classifiable	20 (8.3%)
Primary site *	Extremity/trunk	67 (27.9%)
	Uterus	55 (22.9%)
	Retroperitoneum	54 (22.5%)
	Non-uterine abdomen	50 (20.8%)
	Head and neck	13 (5.4%)
Grade, evaluable *n* = 216 †	G1	16 (7.4%)
	G2	65 (30.1%)
	G3	135 (62.5%)

* 1 patient was not evaluable for the primary site; † grade was evaluable in 216 of 240 patients (90.0%).

**Table 2:** Overall survival according to disease stage, primary site, histologic grade, and age group.

Comparison	Group	*n*	Events	Median OS, Months (95% CI)	5-Year OS, % (95% CI)	Log-Rank *p*
Overall	All patients	240	131	37.0 (26.0–48.0)	33.9% (25.9–42.0%)	—
Stage †	Early/localized	132	47	61.0 (43.9–78.1)	50.7% (37.6–62.4%)	<0.001
	Metastatic	88	68	13.0 (10.2–15.8)	8.8% (2.8–19.1%)	
Disease status	Localized	120	41	61.1 (52.3–97.4)	51.2% (37.1–63.7%)	<0.001 #
	Recurrent localized	12	6	58.1 (9.3–NR)	41.7% (12.0–69.6%)	
	Recurrent metastatic	35	22	19.8 (9.9–44.9)	20.4% (6.1–40.5%)	
	De novo metastatic	53	46	11.7 (7.0–15.7)	3.3% (0.3–13.1%)	
Age	<50 years	78	35	51.0 (27.1–74.9)	44.6% (29.5–58.6%)	0.017
	≥50 years	162	96	28.0 (17.1–38.9)	28.8% (19.6–38.6%)	
Extremity comparison *	Extremity/trunk	67	31	53.0 (22.4–83.6)	47.0% (30.9–61.5%)	0.040
	Non-extremity	172	99	28.0 (17.0–39.0)	29.0% (20.2–38.4%)	
Uterine comparison *	Uterine	55	33	28.0 (12.3–43.7)	30.8% (16.8–46.0%)	0.401
	Non-uterine	184	97	39.0 (22.4–55.6)	34.6% (25.2–44.2%)	
Grade ‡	Grade 1/2	81	31	61.1 (52.3–NR)	54.5% (38.2–68.1%)	<0.001
	Grade 3	135	84	25.6 (18.1–37.4)	28.0% (18.8–37.9%)	

CI, confidence interval; OS, overall survival; NR, not reached. * One patient had no primary site ascertained and was therefore excluded from the anatomical-site analyses; these analyses included 239 patients. † Stage-specific analyses included 220 patients, with 115 total events among stage-evaluable patients. ‡ Grade-specific analysis included 216 patients (90.0% of the cohort), with 115 total events among grade-evaluable patients. # Overall, 4-group log-rank *p* < 0.001.

**Table 3:** Multivariable Cox proportional hazards models evaluating the two anatomical site comparisons.

Covariate	Extremity/Trunk Model HR (95% CI)	*p*	Uterine Model HR (95% CI)	*p*
Age ≥ 50 vs. <50	1.86 (1.19–2.91)	0.006	1.92 (1.23–3.01)	0.004
Male vs. Female	1.09 (0.70–1.71)	0.70	0.93 (0.58–1.49)	0.75
Grade 3 vs. grade 1/2	1.92 (1.20–3.09)	0.007	1.74 (1.10–2.76)	0.018
Metastatic vs. early stage	5.59 (3.55–8.80)	<0.001	5.94 (3.79–9.33)	<0.001
Non-extremity vs. extremity/trunk	1.60 (0.97–2.63)	0.063	—	—
Non-uterine vs. uterine	—	—	1.23 (0.75–2.03)	0.41

CI, confidence interval; HR, hazard ratio; *N* = 197 for both models (patients with missing histologic grade and/or unclassifiable stage excluded); 100 events. Extremity/trunk, uterine, and grade 1/2 were used as reference categories throughout.

**Table 4:** Extent of missing data for key clinical variables.

Variable	Evaluable, *n*/*N*	Missing, *n* (%)
Disease stage	220/240	20 (8.3%)
Primary site	239/240	1 (0.4%)
Histologic grade	216/240	24 (10.0%)
Tumor size	204/240	36 (15.0%)
Surgery (early/localized, *n* = 132)	127/132	5 (3.8%)
Neoadjuvant chemotherapy (early/localized)	118/132	14 (10.6%)
Adjuvant chemotherapy (early/localized)	112/132	20 (15.2%)
Adjuvant radiotherapy (early/localized)	107/132	25 (18.9%)
Recurrence status (early/localized)	93/132	39 (29.5%)
Systemic therapy (metastatic, *n* = 88)	79/88	9 (10.2%)
Metastasectomy (metastatic)	68/88	20 (22.7%)
Oligometastatic status (metastatic, *n* = 88)	79/88	9 (10.2%)

## Data Availability

The data supporting the findings of this study are available from the corresponding author upon reasonable request. The data are not publicly available because they contain clinical information derived from individual patient medical records.

## Supplementary Materials

The following supporting information can be downloaded at: https://www.mdpi.com/article/10.3390/jcm15197693/s1. Supplementary Table S1. Treatment patterns according to disease status; Supplementary Table S2. Metastatic disease rates according to anatomical-site grouping*; Supplementary Table S3. Multivariable Cox proportional hazards models evaluating the association of anatomical site with overall survival, with age modeled continuously as a sensitivity analysis; Supplementary Table S4. Comparison of overall survival outcomes across published leiomyosarcoma cohorts and the present study; Supplementary Table S5. Early/localized disease (*n* = 132): baseline vs. treatment-inclusive multivariable Cox models; Supplementary Table S6. Metastatic disease (*n* = 88): baseline vs. treatment-inclusive multivariable Cox models; Supplementary Figure S1. Distribution of metastatic sites among evaluable metastatic patients; Supplementary Figure S2. Kaplan-Meier overall survival by uterine versus non-uterine primary site; Supplementary Figure S3. Distribution of disease stage according to primary anatomical site; Supplementary Analysis S1. Treatment-Inclusive and Stage-Stratified Sensitivity Analyses.
