# Supplementary material for: Clinical Heterogeneity and Survival Disparities in Leiomyosarcoma: An Exploratory Single-Center Retrospective Observational Study
Source: J Clin Med. 2026 Oct 4;15(19):7693. doi: 10.3390/jcm15197693 (PMC13666196; doi:10.3390/jcm15197693)
Supplement: Supplementary file 1 [file jcm-15-07693-s001.zip › jcm-4554615-supplementary.pdf]

Supplementary Material

Clinical Heterogeneity and Survival Disparities in Leiomyosarcoma: A Real-World Cohort Study

All models presented in Supplementary Tables S1–S4 correspond to the analyses described in the main manuscript Methods and Results.

Supplementary Table S1. Treatment patterns according to disease status.

Treatment characteristic	Early/localized	Metastatic
Patients	132	88
Surgery	119/127 (93.7%)	—
Neoadjuvant chemotherapy	6/118 (5.1%)	—
Adjuvant chemotherapy	32/112 (28.6%)	—
Adjuvant radiotherapy	26/107 (24.3%)	—
Recurrence	40/93 (43.0%)	—
Systemic therapy	—	67/79 (84.8%)
Metastasectomy	—	9/68 (13.2%)

Denominators varied across treatment variables because of missing or unavailable documentation.

Supplementary Table S2. Metastatic disease rates according to anatomical-site grouping*

Anatomical-site grouping	Early/localized, n	Metastatic, n	Total, n	Metastatic rate	Statistical test
	Uterine vs non-uterine				χ^2=4.71, P=.030; Fisher's exact P=.024
Uterine	24	28	52	53.8%	
Non-uterine	108	60	168	35.7%	
	Extremity/trunk vs non-extremity				χ^2=1.73, P=.188; Fisher's exact P=.169
Extremity/trunk	42	20	62	32.3%	
Non-extremity	90	68	158	43.0%	

**Cross-tabulations exclude 20 patients with unclassifiable stage at presentation. Tests are two-sided chi-square with Fisher's exact test shown for confirmation given the smaller cell counts in the uterine comparison.*

Supplementary Table S3. Multivariable Cox proportional hazards models evaluating the association of anatomical site with overall survival, with age modeled continuously as a sensitivity analysis

N=197, 100 events in all four models (patients with missing histologic grade and/or unclassifiable stage excluded).

Covariate	Extremity model Continuous age	Extremity model Age ≥50	Uterine model Continuous age	Uterine model Age ≥50
Age	HR 1.035/year (95% CI, 1.016–1.054), P<.001	HR 1.86 (95% CI, 1.19–2.91), P=.006	HR 1.034/year (95% CI, 1.016–1.053), P<.001	HR 1.92 (95% CI, 1.23–3.01), P=.004
Male vs female	1.10 (95% CI, 0.71–1.72), P=.67	1.09 (95% CI, 0.70–1.71), P=.70	0.95 (95% CI, 0.59–1.53), P=.84	0.93 (95% CI, 0.58–1.49), P=.75
Grade 3 vs grade 1/2	1.93 (95% CI, 1.21–3.08), P=.006	1.92 (95% CI, 1.20–3.09), P=.007	1.79 (95% CI, 1.13–2.82), P=.013	1.74 (95% CI, 1.10–2.76), P=.018
Metastatic vs early	6.35 (95% CI, 3.96–10.18), P<.001	5.59 (95% CI, 3.55–8.80), P<.001	6.66 (95% CI, 4.18–10.63), P<.001	5.94 (95% CI, 3.79–9.33), P<.001
Site comparison	Non-extremity vs extremity/trunk: 1.59 (95% CI, 0.97–2.59), P=.064	Non-extremity vs extremity/trunk: 1.60 (95% CI, 0.97–2.63), P=.063	Non-uterine vs uterine: 1.23 (95% CI, 0.75–2.01), P=.42	Non-uterine vs uterine: 1.23 (95% CI, 0.75–2.03), P=.41

Hazard ratios (HRs) with 95% confidence intervals (CIs) were estimated using multivariable Cox proportional hazards regression. Separate models evaluated extremity/trunk and uterine site as the anatomical-site variable. Models were fitted using age as a continuous variable and, separately, using age categorized as ≥50 years versus <50 years. All models were adjusted for sex, histologic grade (grade 3 vs grade 1/2), and stage at presentation (metastatic vs early-stage disease). Extremity/trunk and uterine site were used as reference categories. HRs >1 indicate a higher hazard of death.

Supplementary Table S4. Comparison of overall survival outcomes across published leiomyosarcoma cohorts and the present study

Series	N	Design	Site	Median OS	5-year OS
Present cohort	240	Retrospective, single-centre	Mixed	37.0 months (95% CI, 26.0–48.0)	33.9% (95% CI, 25.9%–42.0%)
Yang et al. 2024 [4]	Review	Review	Uterine	—	25–76% overall; 10–15% if metastatic
Ikoma et al. 2017 [8]	85	Retrospective, single-centre; resected	Retroperitoneal	8.3 years (95% CI 5.7–12.3)	—
Øines et al. 2024 [17]	Pooled	Systematic review	Abdomen/retroperitoneum vs other sites	—	10-year OS: 63.4% vs 79.2%
Ullah et al. 2024 [18]	1,406	Retrospective; JCO abstract	Retroperitoneal	—	40%
Kapp et al. 2008 [11]	1,396	Retrospective registry	Uterine	—	91.4%, 74.2%, and 57.4% for grade 1, 2, and 3, respectively
Xue et al. 2019 [5]	1,528	Retrospective registry	Extremity	105 months	61.7%

OS, overall survival; NR, not reached; CI, confidence interval.

Supplementary Figure S1. Distribution of metastatic sites among evaluable metastatic patients.

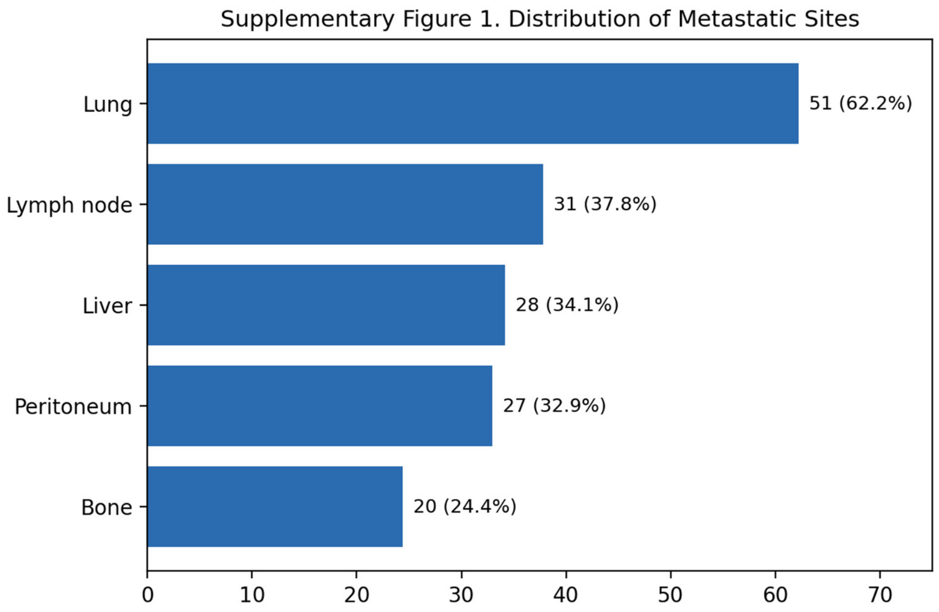

Supplementary Figure S2. Kaplan-Meier overall survival by uterine versus non-uterine primary site.

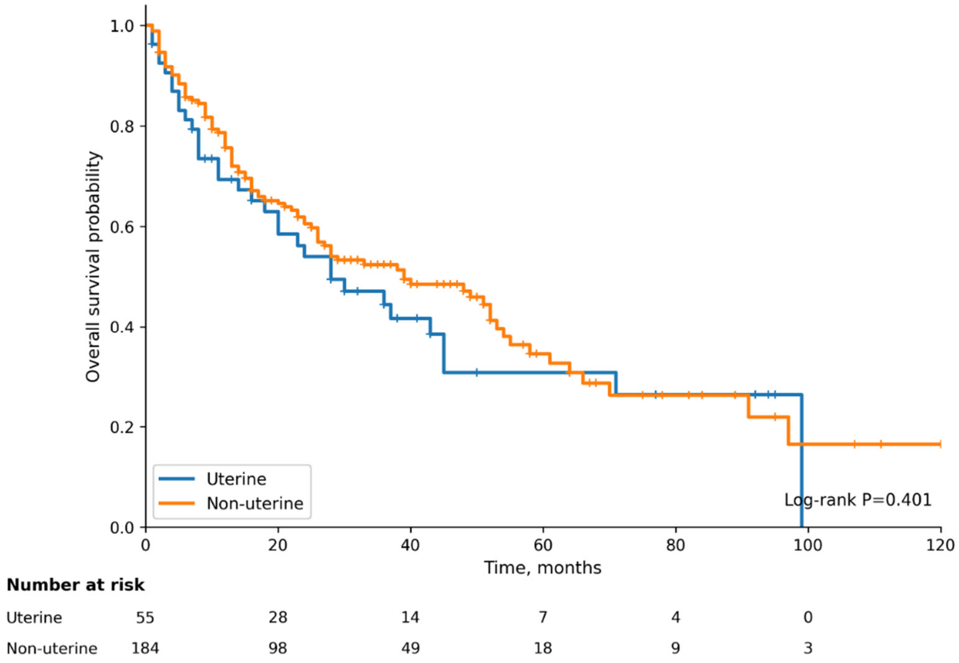

Supplementary Figure S3. Distribution of disease stage according to primary anatomical site.

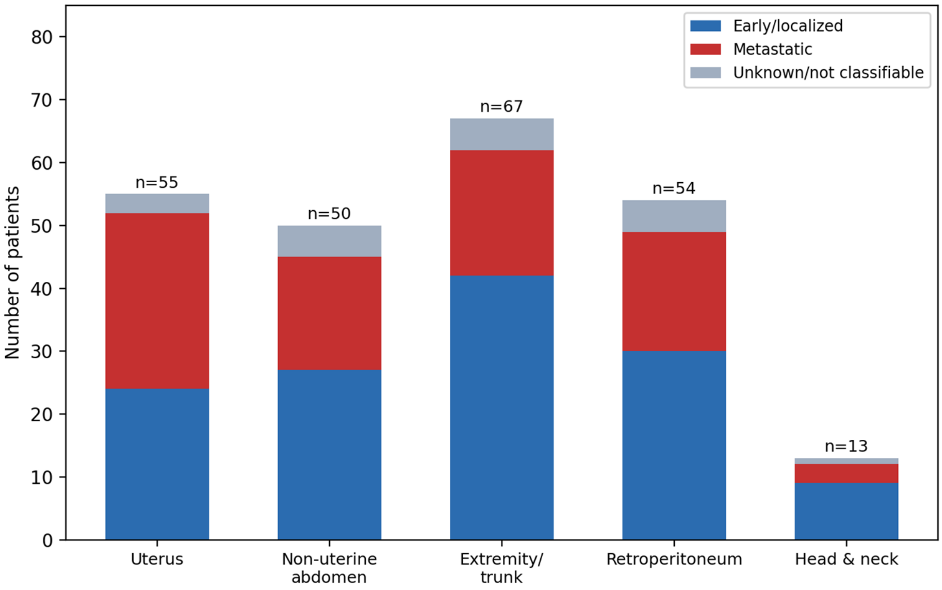

Supplementary Analysis S1. Treatment-Inclusive and Stage-Stratified Sensitivity Analyses

To further examine the association of treatment variables with overall survival, treatment-inclusive Cox proportional hazards models were evaluated separately within early/localized and metastatic disease. Each model retained age, sex, histologic grade, and the corresponding anatomical-site comparison used in the primary analysis, with treatment variables relevant to the respective disease setting added as exploratory covariates.

Early/localized disease. The treatment-inclusive model incorporated age, sex, histologic grade, anatomical site, surgery, adjuvant chemotherapy, and adjuvant radiotherapy and included 99 patients with 30 events. Surgery was independently associated with longer OS in both anatomical-site models (HR, 0.11; 95% CI, 0.02–0.67; P=.016 in the extremity-site model; HR, 0.13; 95% CI, 0.02–0.75; P=.022 in the uterine-site model). Neither adjuvant chemotherapy nor adjuvant radiotherapy was independently associated with OS. Anatomical site was not independently associated with OS in either model (non-extremity vs extremity/trunk: HR, 0.96; P=.94; non-uterine vs uterine: HR, 0.76; P=.64).

To examine the relationship between surgery and the prognostic association of histologic grade, surgery was subsequently removed while age, sex, grade, anatomical site, adjuvant chemotherapy, and adjuvant radiotherapy were retained. In this otherwise identical model, grade 3 histology was independently associated with inferior OS in both anatomical-site models (HR, 2.80; 95% CI, 1.05–7.44; P=.039 in the extremity-site model; HR, 2.83; 95% CI, 1.14–7.00; P=.024 in the uterine-site model). Neither adjuvant chemotherapy nor adjuvant radiotherapy was independently associated with OS. The attenuation of the grade association after inclusion of surgery suggests that adjustment for surgery accounts for part of the observed prognostic association of grade with survival; this finding should be interpreted in the context of the observational treatment allocation. (see Supplementary Analysis A1)

Metastatic disease. The treatment-inclusive model incorporated age, sex, histologic grade, anatomical site, surgery, systemic therapy, and metastasectomy and included 59 patients with 44 events. Metastasectomy was independently associated with longer OS in both anatomical-site models (HR, 0.21; 95% CI, 0.05–0.93; P=.039 in the extremity-site model; HR, 0.25; 95% CI, 0.06–0.96; P=.043 in the uterine-site model). Surgery and systemic therapy were not independently associated with OS. Anatomical site was not independently associated with OS in either model. (see Supplementary Analysis A1)

Overall, these stage-stratified treatment-inclusive analyses provided consistent evidence that treatment associations differed according to disease setting, while anatomical site remained non-significantly associated with OS after adjustment for treatment variables and other

clinical covariates. Because treatment allocation was non-random and treatment exposures were post-diagnosis variables, the treatment-associated estimates are considered exploratory and should not be interpreted as causal treatment effects.

Supplementary Table S5. Early/localized disease (n=132): baseline vs. treatment-inclusive multivariable Cox models.

Covariate	Baseline model—Extremity HR (95% CI), P	Treatment-inclusive—Extremity HR (95% CI), P	Baseline model—Uterine HR (95% CI), P	Treatment-inclusive—Uterine HR (95% CI), P
Age ≥50 vs <50	1.65 (0.81–3.38), .169	2.28 (0.94–5.50), .068	1.57 (0.75–3.28), .230	2.45 (0.95–6.27), .063
Male vs Female	1.32 (0.63–2.78), .465	1.16 (0.50–2.73), .729	1.04 (0.50–2.16), .913	1.30 (0.51–3.28), .585
**Grade 3 vs grade 1/2**	**3.61 (1.58–8.24), .002**	**2.36 (0.86–6.43), .094**	**2.71 (1.30–5.66), .008**	**2.53 (1.00–6.38), .049**
**Surgery (yes vs no)**	—	**0.11 (0.02–0.67), .016**	—	**0.13 (0.02–0.75), .022**
Adjuvant chemotherapy	—	1.49 (0.63–3.54), .363	—	1.39 (0.58–3.29), .460
Adjuvant radiotherapy	—	0.43 (0.15–1.22), .114	—	0.40 (0.13–1.20), .102
Site (non-extremity vs extremity/trunk / non-uterine vs uterine)	1.94 (0.88–4.28), .100	0.96 (0.37–2.50), .938	0.89 (0.38–2.11), .794	0.76 (0.25–2.37), .641

N=121, 40 events (baseline models); N=99, 30 events (treatment-inclusive models). Reference categories: age <50, female, grade 1/2, extremity/trunk or uterine site, no surgery, no adjuvant chemotherapy, no adjuvant radiotherapy.

Supplementary Table S6. Metastatic disease (n=88): baseline vs. treatment-inclusive multivariable Cox models.

Covariate	Baseline model—Extremity HR (95% CI), P	Treatment-inclusive—Extremity HR (95% CI), P	Baseline model—Uterine HR (95% CI), P	Treatment-inclusive—Uterine HR (95% CI), P
**Age ≥50 vs <50**	**1.90 (1.08–3.33), .026**	**1.51 (0.75–3.01), .247**	**2.07 (1.16–3.67), .014**	**1.68 (0.86–3.28), .131**
Male vs Female	1.06 (0.59–1.89), .846	0.86 (0.41–1.78), .673	0.82 (0.44–1.54), .533	0.73 (0.34–1.58), .429
Grade 3 vs grade 1/2	1.35 (0.75–2.40), .315	1.59 (0.76–3.35), .218	1.42 (0.79–2.54), .241	1.60 (0.77–3.36), .209
Surgery (yes vs no)	—	0.61 (0.29–1.26), .180	—	0.69 (0.31–1.54), .363

Covariate	Baseline model—Extremity HR (95% CI), P	Treatment-inclusive—Extremity HR (95% CI), P	Baseline model—Uterine HR (95% CI), P	Treatment-inclusive—Uterine HR (95% CI), P
Systemic therapy	—	0.41 (0.10–1.62), .205	—	0.43 (0.11–1.66), .223
**Metastasectomy**	**—**	**0.21 (0.05–0.93), .039**	**—**	**0.25 (0.06–0.96), .043**
Site (non-extremity vs extremity/trunk / non-uterine vs uterine)	1.29 (0.66–2.50), .459	0.70 (0.29–1.66), .413	1.73 (0.92–3.22), .087	1.56 (0.66–3.68), .315
N=76, 60 events (baseline models); N=59, 44 events (treatment-inclusive models). Reference categories: age <50, female, grade 1/2, extremity/trunk or uterine site, no surgery, no systemic therapy, no metastasectomy.				

CI, confidence interval; HR, hazard ratio. Site is not independently associated with overall survival in any baseline or treatment-inclusive model, in either stratum. Grade 3 attenuates from significant to borderline/non-significant in early/localized disease once surgery is added, consistent with surgery mediating part of grade's prognostic effect.
